# Supplementary material for: Associations between future health expectations and patient satisfaction after lumbar spine surgery: a longitudinal observational study of 9929 lumbar spine surgery procedures
Source: BMJ Open. 2023 Sep 25;13(9):e074072. doi: 10.1136/bmjopen-2023-074072 (PMC10533696; doi:10.1136/bmjopen-2023-074072)
Supplement: Supplementary data [file bmjopen-2023-074072supp001.pdf]

Table S1 Comparison of baseline characteristics between included and excluded patients.

	LSS		LDH	
	Included	*Excluded*	*Included*	*Excluded*
n	3969	5639	5960	8559
Age, mean (SD)	66 (10.1)	67.8 (10.8)	44.8 (12.6)	44.4 (13.1)
BMI, mean (SD)	27.6 (4.01)	27.7 (4.18)	26.3 (4.12)	26.6 (4.28)
Women, n (%)	1819 (45.8)	2855 (50.6)	2635 (44.2)	3760 (43.9)

Table S2 Distribution of the SF-36 item 11c (I expect my health to get worse) responses preoperatively and year 1 for LSS (n=3969) and LDH (n=5960).

		1. Definitely True	2. Mostly True	3. Don't know	4. Mostly False	5. Definitely False
LSS, n (%)	*Preop*	78 (1.97)	226 (5.69)	1197 (30.2)	935 (23.6)	1533 (38.6)
	Year 1	157 (3.96)	489 (12.3)	1471 (37.1)	724 (18.2)	1128 (28.4)
LDH, n (%)	*Preop*	93 (1.56)	206 (3.46)	1034 (17.3)	1307 (21.9)	3320 (55.7)
	Year 1	137 (2.3)	427 (7.16)	1483 (24.9)	1270 (21.3)	2643 (44.3)

Table S3 Preoperative and year one postoperative SF-36 data for patients treated surgically for one-level central spinal stenosis between 2007 to 2016.

	Negative future health expectations preoperatively			Positive future health expectations preoperatively		
	Preop Mean (95% CI)	*Year one Mean (95% CI)*	*SRM (95% CI)*	*Preop Mean (95% CI)*	*Year one Mean (95% CI)*	*SRM (95% CI)*
PF	35 (34;36)	54 (53;55)	0.78 (0.72;0.83)	41 (40;42)	67 (66;68)	1.06 (1.01;1.11)
RP	11 (9.7;12)	38 (36;40)	0.62 (0.57;0.67)	17 (16;18)	57 (56;59)	0.87 (0.82;0.92)
BP	28 (27;28)	50 (49;51)	0.86 (0.82;0.91)	30 (29;31)	60 (59;61)	1.08 (1.04;1.12)
GH	48 (47;48)	53 (51;54)	0.27 (0.22;0.32)	69 (69;70)	69 (68;69)	-0.024 (-0.063;0.015)
VT	35 (34;36)	49 (47;50)	0.59 (0.54;0.64)	44 (44;45)	61 (60;62)	0.68 (0.64;0.72)
SF	53 (52;54)	71 (69;72)	0.61 (0.56;0.67)	63 (62;64)	82 (81;83)	0.66 (0.62;0.71)
RE	35 (33;37)	57 (55;59)	0.42 (0.37;0.47)	52 (50;54)	75 (74;77)	0.46 (0.42;0.5)
MH	61 (60;62)	70 (69;71)	0.45 (0.4;0.5)	71 (70;72)	80 (79;81)	0.44 (0.4;0.48)

Table S4 Preoperative and year one postoperative SF-36 data for patients treated surgically for one-level disk herniation between 2007 to 2016.

	Negative future health expectations preoperatively			Positive future health expectations preoperatively		
	Preop Mean (95% CI)	*Year one Mean (95% CI)*	*SRM (95% CI)*	*Preop Mean (95% CI)*	*Year one Mean (95% CI)*	*SRM (95% CI)*
PF	37 (36;38)	71 (69;72)	1.2 (1.1;1.3)	41 (41;42)	81 (81;82)	1.54 (1.49;1.58)
RP	8.9 (7.8;10)	53 (51;56)	0.99 (0.93;1.1)	10 (9.7;11)	69 (68;71)	1.37 (1.32;1.41)
BP	20 (19;21)	56 (55;58)	1.3 (1.2;1.3)	23 (22;23)	67 (66;67)	1.55 (1.51;1.59)
GH	49 (48;50)	57 (56;59)	0.39 (0.34;0.45)	74 (74;75)	76 (75;76)	0.076 (0.048;0.11)
VT	28 (27;29)	51 (49;52)	0.88 (0.81;0.94)	35 (35;36)	64 (63;64)	1.1 (1.1;1.1)
SF	42 (41;44)	75 (74;77)	1 (0.97;1.1)	49 (48;50)	87 (86;87)	1.2 (1.1;1.2)
RE	34 (32;36)	68 (66;70)	0.67 (0.61;0.73)	50 (49;52)	83 (82;84)	0.66 (0.62;0.69)
MH	52 (51;53)	69 (68;70)	0.74 (0.68;0.79)	64 (63;65)	81 (80;81)	0.79 (0.75;0.82)

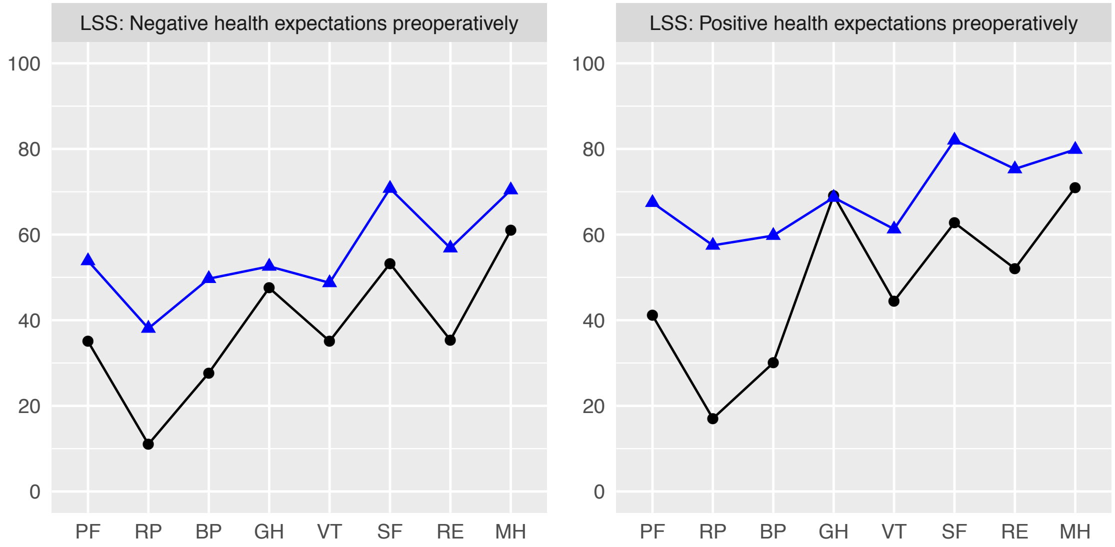

Figure S1 SF-36 profiles preoperatively (black circles) and year one postoperatively (blue triangles) for patients with negative and positive future health expectations preoperatively treated for LSS.
PF = physical functioning, RP = role limitation due to physical problems, BP = bodily pain,
GH = general health, VT = vitality, SF = social functioning, RE = role limitations due to emotional problems, and MH = mental health.

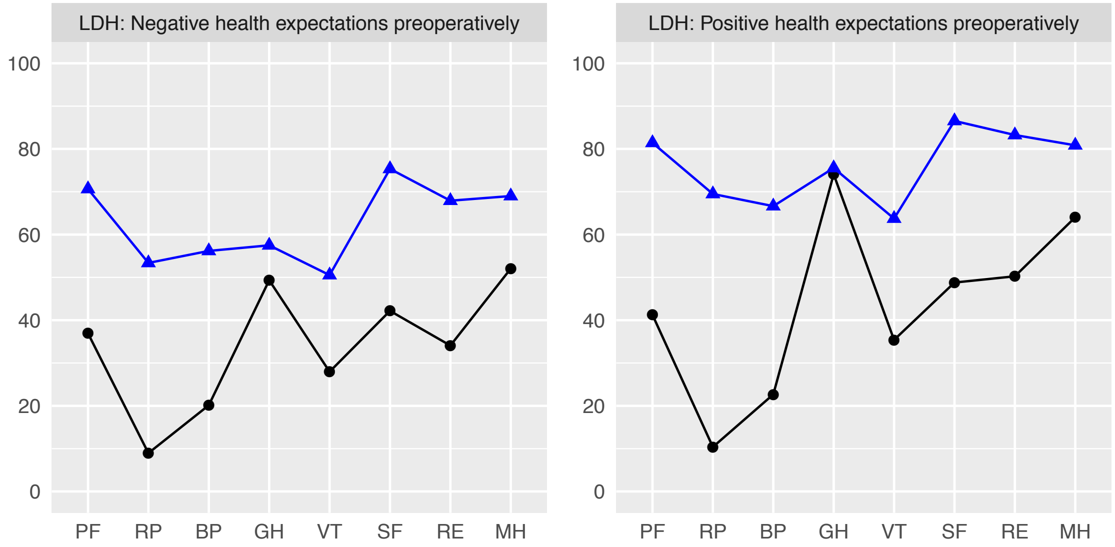

Figure S2 SF-36 profiles preoperatively (black circles) and year one postoperatively (blue triangles) for patients with negative and positive future health expectations preoperatively treated for LDH.
PF = physical functioning, RP = role limitation due to physical problems, BP = bodily pain, GH = general health, VT = vitality, SF = social functioning, RE = role limitations due to emotional problems, and MH = mental health.
