# Supplementary material for: Associations between future health expectations and patient satisfaction after lumbar spine surgery: a longitudinal observational study of 9929 lumbar spine surgery procedures
Source: BMJ Open. 2023 Sep 25;13(9):e074072. doi: 10.1136/bmjopen-2023-074072 (PMC10533696; doi:10.1136/bmjopen-2023-074072)
Supplement: Author's manuscript [file bmjopen-2023-074072.draft_revisions.pdf]

BMJ Open is committed to open peer review. As part of this commitment we make the peer review history of every article we publish publicly available.

When an article is published we post the peer reviewers' comments and the authors' responses online. We also post the versions of the paper that were used during peer review. These are the versions that the peer review comments apply to.

The versions of the paper that follow are the versions that were submitted during the peer review process. They are not the versions of record or the final published versions. They should not be cited or distributed as the published version of this manuscript.

BMJ Open is an open access journal and the full, final, typeset and author-corrected version of record of the manuscript is available on our site with no access controls, subscription charges or pay-per-view fees (http://bmjopen.bmj.com).

If you have any questions on BMJ Open's open peer review process please email info.bmjopen@bmj.com

BMJ Open

Associations between future health expectations and patient satisfaction after lumbar spine surgery: Observations from 9929 lumbar spine surgery procedures

Journal:	*BMJ Open*
Manuscript ID	bmjopen-2023-074072
Article Type:	Original research
Date Submitted by the Author:	26-Mar-2023
Complete List of Authors:	Joelson, Anders; Orebro universitet<br>Szigethy, Lilla; Region Orebro lan<br>Wildeman, Peter; Orebro universitet<br>Sigmundsson, Freyr Gauti; Orebro universitet<br>Karlsson, Jan; Orebro universitet
Keywords:	Spine < ORTHOPAEDIC & TRAUMA SURGERY, Adult orthopaedics < ORTHOPAEDIC & TRAUMA SURGERY, Quality of Life

SCHOLARONE™
Manuscripts

I, the Submitting Author has the right to grant and does grant on behalf of all authors of the Work (as defined in the below author licence), an exclusive licence and/or a non-exclusive licence for contributions from authors who are: i) UK Crown employees; ii) where BMJ has agreed a CC-BY licence shall apply, and/or iii) in accordance with the terms applicable for US Federal Government officers or employees acting as part of their official duties; on a worldwide, perpetual, irrevocable, royalty-free basis to BMJ Publishing Group Ltd ("BMJ") its licensees and where the relevant Journal is co-owned by BMJ to the co-owners of the Journal, to publish the Work in this journal and any other BMJ products and to exploit all rights, as set out in our licence.

The Submitting Author accepts and understands that any supply made under these terms is made by BMJ to the Submitting Author unless you are acting as an employee on behalf of your employer or a postgraduate student of an affiliated institution which is paying any applicable article publishing charge ("APC") for Open Access articles. Where the Submitting Author wishes to make the Work available on an Open Access basis (and intends to pay the relevant APC), the terms of reuse of such Open Access shall be governed by a Creative Commons licence – details of these licences and which Creative Commons licence will apply to this Work are set out in our licence referred to above.

Other than as permitted in any relevant BMJ Author's Self Archiving Policies, I confirm this Work has not been accepted for publication elsewhere, is not being considered for publication elsewhere and does not duplicate material already published. I confirm all authors consent to publication of this Work and authorise the granting of this licence.

For peer review only

Associations between future health expectations and patient satisfaction after lumbar spine surgery: Observations from 9929 lumbar spine surgery procedures

Anders Joelson, MD, PhD[1,2], Lilla Szigethy, MD[2], Peter Wildeman, MD, PhD[1,2], Freyr Gauti Sigmundsson, MD, PhD[1,2], Jan Karlsson, PhD[3]

1. Örebro University School of Medical Sciences, SE-70182 Örebro, Sweden
2. Department of Orthopaedics, Örebro University Hospital, SE-70185 Örebro, Sweden
3. University Health Care Research Center, Faculty of Medicine and Health, Örebro University, SE-70182, Örebro, Sweden

Correspondence:
Anders Joelson
E-mail address: anders.joelson@oru.se
Tel: +46196021000 Fax: +46196025337

For peer review only

Abstract

Objective: This study aimed to investigate the associations between general health expectations and patient satisfaction with treatment for the two common spine surgery procedures diskectomy for lumbar disk herniations (LDH) and decompression for lumbar spinal stenosis (LSS).

Design: Register study with prospectively collected preoperative and one-year postoperative data.

Setting: National outcome data from Swespine, the National Swedish spine register.

Participants: A total of 9929 patients, aged between 20 and 85 years, who were self-reported non-smokers, and were operated between 2007 and 2016 for one-level LSS without degenerative spondylolisthesis, or one-level LDH, were identified in the national Swedish spine register (Swespine). We used SF-36 items 11c and 11d to assess future health expectations and present health perceptions. Satisfaction with treatment was assessed using the Swespine satisfaction item.

Interventions: One-level diskectomy for LDH or one-level decompression for LSS.

Primary outcome measures: Satisfaction with treatment.

Results: For LSS, the year one satisfaction ratio amongst patients with low future health expectations preoperatively was 60% (95% CI 58 to 63), while it was 75% (95% CI 73 to 76) for patients with high future health expectations preoperatively. The corresponding numbers for LDH were 73% (95% CI 71 to 75) and 84% (95% CI 83 to 85) respectively. Multiple linear logistic regression models for patient satisfaction one year postoperatively indicated that future health expectations had a larger impact on patient satisfaction than present health perceptions.

Conclusions: Patients operated for the common lumbar spine diseases LSS or LDH, with low future general health expectations, were significantly less satisfied with treatment than patients with high expectations with regards to future general health. These findings are important for patients, and for the surgeons who counsel them, when surgery is a treatment option for LSS or LDH.

For peer review only

Strengths and limitations

1. To our knowledge, this is the first study to report data on the associations between expectations on future general health assessed preoperatively and patient satisfaction after lumbar spine surgery.

2. The study includes a large number of patients from a national database with high, stable coverage.

3. We recognize the inherent limitations of register data, such as lack of confounder information, missing data, or unknown data quality.

4. The data were incomplete in 58% of the procedures.

For peer review only

Introduction

Lumbar degenerative spine diseases are major causes of pain and disability worldwide [1, 2]. Patient-reported outcome measures (PROMs) and patient satisfaction scales are commonly used to evaluate treatment outcomes after lumbar spine surgery [3]. However, there are inconsistencies between PROM changes and treatment satisfaction when evaluating surgical outcome. For example, Chotai et al. [4] found that 83% of patients were satisfied with treatment after elective surgery for degenerative spine disease, whereas only 62% achieved minimal important change (MIC) for the Oswestry/neck disability indices. Furthermore, Godil et al. [5], in an analysis using receiver operating characteristics (ROC) curves, found that improvement in the Oswestry disability index failed to discriminate between satisfaction and dissatisfaction with treatment with good accuracy after spine surgery. In contrast, Copay et al. [6] found a strong association between the Oswestry disability index and patient satisfaction after lumbar surgery. The variety of results suggest that patient satisfaction is also influenced by factors other than PROM changes, such as expectations, socioeconomic factors, and mental health.

The impact of psychological factors on the outcomes of spine surgery has been thoroughly researched [7, 8, 9, 10,]. In addition, several reports have shown that preoperative expectations on recovery predict the outcome of spine surgery [11, 12, 13, 14, 15, 16, 17, 18]. Iderberg et al. [19] demonstrated that socioeconomic indicators are associated with the outcomes of surgery for lumbar spinal stenosis.

However, there seems to be a knowledge gap regarding whether expectations on future general health are associated with the outcomes of spine surgery. Therefore, the current study aimed to

For peer review only

investigate the associations between future general health expectations and patient satisfaction following surgery for degenerative spine diseases.

Methods

Study design

The present study was a register study, with prospectively collected longitudinal data from Swespine, the national Swedish spine register [20].

The national Swedish spine register (Swespine)

The Swespine register was launched in 1992 and covers 90% of the spine units in Sweden. The one-year follow-up rate is 70-75% [20]. The register includes data on diagnoses, surgical procedures, complications, and patient-reported outcome measures. The surgeon is responsible for submitting data about the surgery.

Patient data set

Patients, aged between 20 and 85 years, who were self-reported non-smokers, and were surgically treated between 2007 and 2016 for one-level lumbar spinal stenosis (LSS) without degenerative spondylolisthesis, or one-level lumbar disk herniation (LDH), were identified in Swespine.

Measures

The SF-36 is an 8-dimensional, 36-item, self-administered HRQoL instrument for the assessment of general HRQoL [21]. The instrument has 6 items for assessment of general health perceptions:

For peer review only

item 1 (present health), item 2 (health transition), item 11a (health comparison), item 11b (health context), item 11c (future health), and item 11d (present health). In our study, we used items 11c and 11d to assess future health expectations and present health perceptions (Table 1). We grouped future health expectations into low health expectations (item 11c response options 1, 2, and 3) and high health expectations (item 11c response options 4 and 5). We grouped present health perceptions into high present health perceptions (item 11d response options 1, 2, and 3) and low present health perceptions (item 11c response options 4 and 5). We used the Swedish translation of SF-36 version 1 in our study [22].

Satisfaction with treatment was assessed using the Swespine satisfaction item (Table 1). In our analysis, we grouped satisfaction with treatment into satisfied (response option 1) and dissatisfied (response options 2 and 3).

Table 1 Questions and response options.

	**Question**	**Response options**
Future health (SF-36 Item 11c)	I expect my health to get worse.	1. Definitely True<br>2. Mostly True<br>3. Don't Know<br>4. Mostly False<br>5. Definitely False
Present health (SF-36 Item 11d)	My health is excellent.	1. Definitely True<br>2. Mostly True<br>3. Don't Know<br>4. Mostly False<br>5. Definitely False
Satisfaction (Swespine)	What is your attitude regarding the outcome of your spine surgery?	1. I am satisfied<br>2. I am uncertain<br>3. I am dissatisfied

For peer review only

Statistics

Data are presented as mean and standard deviation (SD) and/or 95% confidence intervals (CIs). Bootstrapping was used to calculate the CIs [23]. Standardized response mean (SRM) for paired data was used to evaluate effect size [24]. The SRM was interpreted as follows: <0.2 no effect, 0.2 to 0.4 small effect, 0.5 to 0.7 moderate effect, >0.7 large effect [25]. Multiple linear logistic regression analysis was used to model the relationship between the outcome and covariates [26].

Patient and public involvement

The patients and the public were not involved in the design, recruitment, conduct, or dissemination plans of this research.

Results

A total of 24127 surgical procedures for the treatment of the lumbar spine diseases LDH and LSS were included in Swespine between 2007 and 2016. Preoperative or one-year postoperative SF-36 data were incomplete for 14198 (58%) of the procedures which provided 9929 procedures eligible for analysis. The baseline characteristics of the included and excluded patients are presented in Supplementary Table S1.

For LSS, 1501 (38%) of 3969 patients had low future health expectations preoperatively and 2117 (53%) of 3969 patients had low future health expectations at the year one follow-up. For LDH, the corresponding number was 1333 (22%) of 5960 patients preoperatively and 2047 (34%) of 5960 patients at the year one follow-up (Supplementary Table S2).

For peer review only - http://bmjopen.bmj.com/site/about/guidelines.xhtml

The preoperative characteristics of the patients with low and high future health expectations are presented in Table 2. The SF-36 profiles preoperatively and one year postoperatively are shown in Figures 1 and 2 and the effect sizes of changes are shown in Supplementary Tables S3 and S4. For LSS, the satisfaction ratio year one postoperatively amongst patients with low future health expectations preoperatively was 60%, while it was 75% for patients with high future health expectations (Table 3). The corresponding levels for LDH were 73% and 84% respectively. Multiple linear logistic regression models for patient satisfaction one year postoperatively indicated that future health expectations had a larger impact on patient satisfaction than present health perceptions (Table 4).

Table 2 Comparison of characteristics between patients with high and low future health expectations preoperatively.

	**LSS (n=3969)**		**LDH (n=5960)**	
	Low future health expectations	*High future health expectations*	*Low future health expectations*	*High future health expectations*
n (%)	1501 (37.8)	2468 (62.2)	1333 (22.4)	4627 (77.6)
Age, mean (SD)	67.8 (10)	64.9 (10.1)	46.8 (13.8)	44.3 (12.1)
BMI, mean (SD)	27.7 (4.0)	27.5 (4.0)	26.7 (4.4)	26.1 (4.0)
Women, n (%)	709 (47.2)	1110 (45)	596 (44.7)	2039 (44.1)

Table 3 Patient satisfaction one year postoperatively for LSS (n=3969) and LDH (n=5960).

	**Low future health expectations preoperatively**	**High future health expectations preoperatively**
LSS, % satisfied (95% CI) (n/total)	60.4 (57.8;62.8) (906/1501)	74.5 (72.9;76.1) (1839/2468)
LDH, % satisfied (95% CI) (n/total)	73 (70.7;75.2) (973/1333)	83.8 (82.8;84.9) (3879/4627)

For peer review only

Table 4 Multiple linear logistic regression models for patient satisfaction one year postoperatively for LSS (n=3969) and LDH (n=5960).

	**LSS**	**LDH**
Intercept, OR (95% CI)	0.981 (0.834;1.15)	1.9 (1.6;2.25)
Future health, OR (95% CI)	1.71 (1.48;1.97)	1.72 (1.48;1.99)
Present health, OR (95% CI)	1.42 (1.23;1.64)	1.47 (1.28;1.69)
Age, OR (95% CI)	1.38 (1.2;1.59)	1.14 (0.994;1.3)
Gender, OR (95% CI)	1.13 (0.98;1.29)	1.17 (1.02;1.33)
BMI, OR (95% CI)	1.25 (1.09;1.44)	1.14 (0.998;1.3)

For peer review only

Discussion

In this paper, we found that patients, operated for the common lumbar spine diseases LSS or LDH, with low future general health expectations preoperatively, were significantly less satisfied with treatment compared with patients with high expectations with regards to future general health. To our knowledge, our study is the first to report data on the association between expectations on future general health assessed preoperatively and patient satisfaction after lumbar spine surgery.

Belayneh et al. [18] studied the impact of future health expectations on the outcome after surgical repair of proximal humeral fractures and found that patients with high expectations on their health, early following the injury, had better long-term outcomes. The authors evaluated future health expectations using a question with exactly the same wording as used in our study. This strengthens the assumption that SF-36 item 11c may be used to assess future health expectations in the field of orthopedic surgery. We agree with the authors that health care providers should communicate with patients, to ensure that they are setting clear expectations of the benefits and risks for each patient.

Iversen et al. [11] studied several expectations summed across the domains pain reduction, physical functioning, and social functioning to evaluate the prognostic importance of preoperative expectations on the treatment outcomes of LSS surgery and found that patients' expectations influence recovery from surgery at six months. The authors concluded that clinicians should discuss expectations with patients preoperatively in order to establish realistic goals and to enable patients to actively engage in their rehabilitation, a conclusion that we agree with.

Standard surgical procedures for the treatment of LDH and LSS are considered safe and beneficial treatment options [27, 28]. However, there are rare but serious complications such as nerve rot lesions [28]. As the main goal of elective surgery for LDH and LSS is to improve patient quality of life, it is important to weigh the benefits against the potential risks when discussing treatment options with patients. For patients with low future health expectations, our data suggests that the satisfaction rate for LSS surgery could be as low as 60%. This information is important from a shared decision-making perspective when balancing the benefits and risks of surgery.

We used SF-36 item 11c to assess future health expectations. Previous studies have indicated that the wording of item 11c sometimes is seen to be unnecessarily negative [29]. Furthermore, Shaples et al. [30] speculated that elderly people might be reluctant to consider questions about worsening in health but concluded that the item did not affect the internal consistency of the SF-36 general health (GH) domain. Although there are some concerns about the design of item 11c, we do not expect that these concerns would invalidate the use of item 11c for the assessment of future health expectations.

For peer review only

Several factors may affect future health expectations. The SF-36 health profiles presented in Figures 1 and 2, and Supplementary Tables S3 and S4 indicate that low future health expectations do not only affect the GH domain, as the patients report lower scores on all SF-36 domains. This illustrates that low future health expectations affects several dimensions of HRQoL.

The five-factor model is commonly used in psychology to model different personality traits [31]. The model uses five orthogonal trait dimensions to describe different personalities: neuroticism (N), extraversion (E), openness (O), agreeableness (A), and conscientiousness (C). Chapman et al. [32] studied the influence of the five-factor model personality traits on perceived health using the NEO Five-Factor Inventory (NEO-FFI) [33] and SF-36. Low N scores and high E scores were associated with high future health expectations. However, although the differences in the personality trait scores were statistically significant, the actual differences were small. Hendriks et al. [34] found that patient satisfaction was only marginally associated with personality. Consequently, the influence of different personality traits on health expectations and patient satisfaction remains unclear, and this field may benefit from further research.

Notably, 38% of the patients with LSS had low future health expectations preoperatively whereas 53% had low future health expectations at the year one follow-up. The corresponding levels for LDH were 22% and 34% respectively. One possible explanation is that preoperatively the patients expect an improvement in health because of the forthcoming operation, while at one year after the operation, the patients may be more neutral or pessimistic about future health improvements. This indicates that questions about future health expectations must be interpreted with caution when asked before and after a health intervention.

For peer review only

The results of our multiple linear logistic regression analysis indicated that there was a strong association between general health assessments (present and future) and patient satisfaction after surgery for LDH and LSS. This association was more pronounced for future health expectations than for present health perceptions. Ferrato et al. [35] reported that there are indications that queries about future health expectations are more useful than those about past health changes in mortality predictions. The findings of our study extend these results to apply to patient satisfaction after lumbar spine surgery. Age, gender, and BMI made only minor contributions as predictors of patient satisfaction one year after LDH surgery, whereas age had some impact on satisfaction after surgery for LSS.

Our findings should be evaluated in the light of several limitations. First, we recognize the inherent limitations of register data, such as lack of confounder information, missing data, or unknown data quality [36]. Second, information on co-morbidities that might affect patient satisfaction was lacking. Third, the data were incomplete in 58% of the procedures**.** Fourth, data on socioeconomic factors were lacking. The study of Iderberg et al. [19] demonstrated that socioeconomic indicators were associated with outcomes of surgery for LSS.

Conclusions

Patients surgically treated for the common lumbar spine diseases LSS or LDH, with low future general health expectations, were significantly less satisfied with treatment compared with patients with high expectations on future general health. The findings of this study can be used in the shared decision-making process when surgery is a treatment option for patients with LSS or LDH to establish realistic expectations and to enable patients to actively engage in rehabilitation.

For peer review only

Contributions

All authors designed the study. AJ analyzed the data. All authors interpreted the data. AJ wrote the manuscript with contributions from PW, LS, FGS, and JK. All authors approved the final version of the manuscript.

Funding

This research received no specific grant from any funding agency in the public, commercial, or not-for-profit sectors.

Competing interests

None.

Patient consent

Not applicable.

Ethics approval

The study was approved by the Swedish Ethical Review Authority (registration number: 2020-03557).

Data availability statement

Data are available from the national Swedish spine register (Swespine) after approval by the Swedish Ethical Review Authority and according to the regulations in the General Data Protection Regulation and the Swedish Patient Data Act.

For peer review only

Figure legends

Figure 1 SF-36 profiles preoperatively (black circles) and year one postoperatively (blue triangles) for patients with low and high future health expectations preoperatively treated for LSS. PF = physical functioning, RP = role limitation due to physical problems, BP = bodily pain, GH = general health, VT = vitality, SF = social functioning, RE = role limitations due to emotional problems, and MH = mental health.

Figure 2 SF-36 profiles preoperatively (black circles) and year one postoperatively (blue triangles) for patients with low and high future health expectations preoperatively treated for LDH. PF = physical functioning, RP = role limitation due to physical problems, BP = bodily pain, GH = general health, VT = vitality, SF = social functioning, RE = role limitations due to emotional problems, and MH = mental health.

For peer review only

References

1. Ravindra VM, Senglaub SS, Rattani A, Dewan MC, Härtl R, Bisson E, Park KB, Shrime MG. Degenerative Lumbar Spine Disease: Estimating Global Incidence and Worldwide Volume. Global Spine J. 2018 Dec;8(8):784-794.

2. Hoy D, March L, Brooks P, Blyth F, Woolf A, Bain C, Williams G, Smith E, Vos T, Barendregt J, Murray C, Burstein R, Buchbinder R. The global burden of low back pain: estimates from the Global Burden of Disease 2010 study. Ann Rheum Dis. 2014 Jun;73(6):968-74.

3. Clement RC, Welander A, Stowell C, Cha TD, Chen JL, Davies M, Fairbank JC, Foley KT, Gehrchen M, Hagg O, Jacobs WC, Kahler R, Khan SN, Lieberman IH, Morisson B, Ohnmeiss DD, Peul WC, Shonnard NH, Smuck MW, Solberg TK, Stromqvist BH, Hooff MLV, Wasan AD, Willems PC, Yeo W, Fritzell P. A proposed set of metrics for standardized outcome reporting in the management of low back pain. Acta Orthop. 2015;86(5):523-33.

4. Chotai S, Sivaganesan A, Parker SL, McGirt MJ, Devin CJ. Patient-Specific Factors Associated With Dissatisfaction After Elective Surgery for Degenerative Spine Diseases. Neurosurgery. 2015 Aug;77(2):157-63; discussion 163.

5. Godil SS, Parker SL, Zuckerman SL, Mendenhall SK, Devin CJ, Asher AL, McGirt MJ. Determining the quality and effectiveness of surgical spine care: patient satisfaction is not a valid proxy. Spine J. 2013 Sep;13(9):1006-12.

For peer review only

6. Copay AG, Martin MM, Subach BR, Carreon LY, Glassman SD, Schuler TC, Berven S. Assessment of spine surgery outcomes: inconsistency of change amongst outcome measurements. Spine J. 2010 Apr;10(4):291-6.

7. Sinikallio S, Aalto T, Koivumaa-Honkanen H, Airaksinen O, Herno A, Kröger H, Viinamäki H. Life dissatisfaction is associated with a poorer surgery outcome and depression among lumbar spinal stenosis patients: a 2-year prospective study. Eur Spine J. 2009 Aug;18(8):1187-93.

8. Sinikallio S, Aalto T, Airaksinen O, Lehto SM, Kröger H, Viinamäki H. Depression is associated with a poorer outcome of lumbar spinal stenosis surgery: a two-year prospective follow-up study. Spine (Phila Pa 1976). 2011 Apr;36(8):677-82.

9. Pakarinen M, Vanhanen S, Sinikallio S, Aalto T, Lehto SM, Airaksinen O, Viinamäki H. Depressive burden is associated with a poorer surgical outcome among lumbar spinal stenosis patients: a 5-year follow-up study. Spine J. 2014 Oct;14(10):2392-6.

10. Miller JA, Derakhshan A, Lubelski D, Alvin MD, McGirt MJ, Benzel EC, Mroz TE. The impact of preoperative depression on quality of life outcomes after lumbar surgery. Spine J. 2015 Jan;15(1):58-64.

11. Iversen MD, Daltroy LH, Fossel AH, Katz JN. The prognostic importance of patient pre-operative expectations of surgery for lumbar spinal stenosis. Patient Educ Couns. 1998 Jun;34(2):169-78.

For peer review only

12. de Groot KI, Boeke S, Passchier J. Preoperative expectations of pain and recovery in relation to postoperative disappointment in patients undergoing lumbar surgery. Med Care. 1999 Feb;37(2):149-56.

13. Lutz GK, Butzlaff ME, Atlas SJ, Keller RB, Singer DE, Deyo RA. The relation between expectations and outcomes in surgery for sciatica. J Gen Intern Med. 1999 Dec;14(12):740-4.

14. Toyone T, Tanaka T, Kato D, Kaneyama R, Otsuka M. Patients' expectations and satisfaction in lumbar spine surgery. Spine (Phila Pa 1976). 2005 Dec;30(23):2689-94.

15. Rönnberg K, Lind B, Zoëga B, Halldin K, Gellerstedt M, Brisby H. Patients' satisfaction with provided care/information and expectations on clinical outcome after lumbar disc herniation surgery. Spine (Phila Pa 1976). 2007 Jan;32(2):256-61.

16. Yee A, Adjei N, Do J, Ford M, Finkelstein J. Do patient expectations of spinal surgery relate to functional outcome? Clin Orthop Relat Res. 2008 May;466(5):1154-61.

17. Mannion AF, Junge A, Elfering A, Dvorak J, Porchet F, Grob D. Great expectations: really the novel predictor of outcome after spinal surgery? Spine (Phila Pa 1976). 2009 Jul;34(15):1590-9.

For peer review only

18. Belayneh R, Lott A, Haglin J, Zuckerman J, Egol K. The role of patients' overall expectations of health on outcomes following proximal humerus fracture repair. Orthop Traumatol Surg Res. 2021 Dec;107(8):103043.

19. Iderberg H, Willers C, Borgström F, Hedlund R, Hägg O, Möller H, Ornstein E, Sandén B, Stalberg H, Torevall-Larsson H, Tullberg T, Fritzell P. Predicting clinical outcome and length of sick leave after surgery for lumbar spinal stenosis in Sweden: a multi-register evaluation. Eur Spine J. 2019 06;28(6):1423-1432.

20. Strömqvist B, Fritzell P, Hägg O, Jönsson B, Sandén B, Swedish Society of Spinal Surgeons. Swespine: The Swedish spine register: The 2012 report. Eur Spine J. 2013 Apr;22(4):953-74.

21. Ware JE Jr, Sherbourne CD. The MOS 36-item short-form health survey (SF-36). I. Conceptual framework and item selection. Med Care. 1992 Jun;30(6):473-83.

22. Sullivan M, Karlsson J, Ware JE Jr. The Swedish SF-36 health survey-I. Evaluation of data quality, scaling assumptions, reliability and construct validity across general populations in Sweden. Soc Sci Med. 1995;41(10):1349-58.

23. Bland JM, Altman DG. Statistics Notes: Bootstrap resampling methods. BMJ. 2015 Jun;350:h2622.

24. Fayers PM, Machin D. Quality of life: The assessment, analysis and reporting of patient-reported outcomes. 3rd ed. Chichester: John Wiley and Sons Ltd; 2016.

For peer review only

25. Cohen J. Statistical power analysis for the behavioral sciences. 2nd ed. Hillsdale, NJ: Lawrence Erlbaum Associates; 1988.

26. Altman DG. Practical statistics for medical research. London: Chapman and Hall; 1991.

27. Weinstein JN, Tosteson TD, Lurie JD, Tosteson ANA, Blood E, Hanscom B, Herkowitz H, Cammisa F, Albert T, Boden SD, Hilibrand A, Goldberg H, Berven S, An H, SPORT Investigators. Surgical versus nonsurgical therapy for lumbar spinal stenosis. N Engl J Med. 2008 Feb;358(8):794-810.

28. Weinstein JN, Lurie JD, Tosteson TD, Tosteson ANA, Blood EA, Abdu WA, Herkowitz H, Hilibrand A, Albert T, Fischgrund J. Surgical versus nonoperative treatment for lumbar disc herniation: four-year results for the Spine Patient Outcomes Research Trial (SPORT). Spine (Phila Pa 1976). 2008 Dec;33(25):2789-800.

29. Hayes V, Morris J, Wolfe C, Morgan M. The SF-36 health survey questionnaire: is it suitable for use with older adults? Age Ageing. 1995 Mar;24(2):120-5.

30. Sharples LD, Todd CJ, Caine N, Tait S. Measurement properties of the Nottingham health profile and short form 36 health status measures in a population sample of elderly people living at home: Results from ELPHS. Br J Health Psychol. 2000;5:217-233.

For peer review only

31. Goldberg LR. The structure of phenotypic personality traits. Am Psychol. 1993 Jan;48(1):26-34.

32. Chapman BP, Duberstein PR, Sörensen S, Lyness JM. Personality and perceived health in older adults: the five factor model in primary care. J Gerontol B Psychol Sci Soc Sci. 2006 Nov;61(6):P362-5.

33. McCrae RR, Costa PT Jr. A contemplated revision of the NEO Five-Factor Inventory. Pers Individ Dif. 2004 FEB;36(3):587-596.

34. Hendriks A, Smets E, Vrielink M, Van Es S, De Haes J. Is personality a determinant of patient satisfaction with hospital care? int j qual health care. 2006 APR;18(2):152-158.

35. Ferraro KF, Wilkinson LR. Alternative Measures of Self-Rated Health for Predicting Mortality Among Older People: Is Past or Future Orientation More Important? Gerontologist. 2015 Oct;55(5):836-44.

36. Thygesen LC, Ersbøll AK. When the entire population is the sample: strengths and limitations in register-based epidemiology. Eur J Epidemiol. 2014 Aug;29(8):551-8.

For peer review only

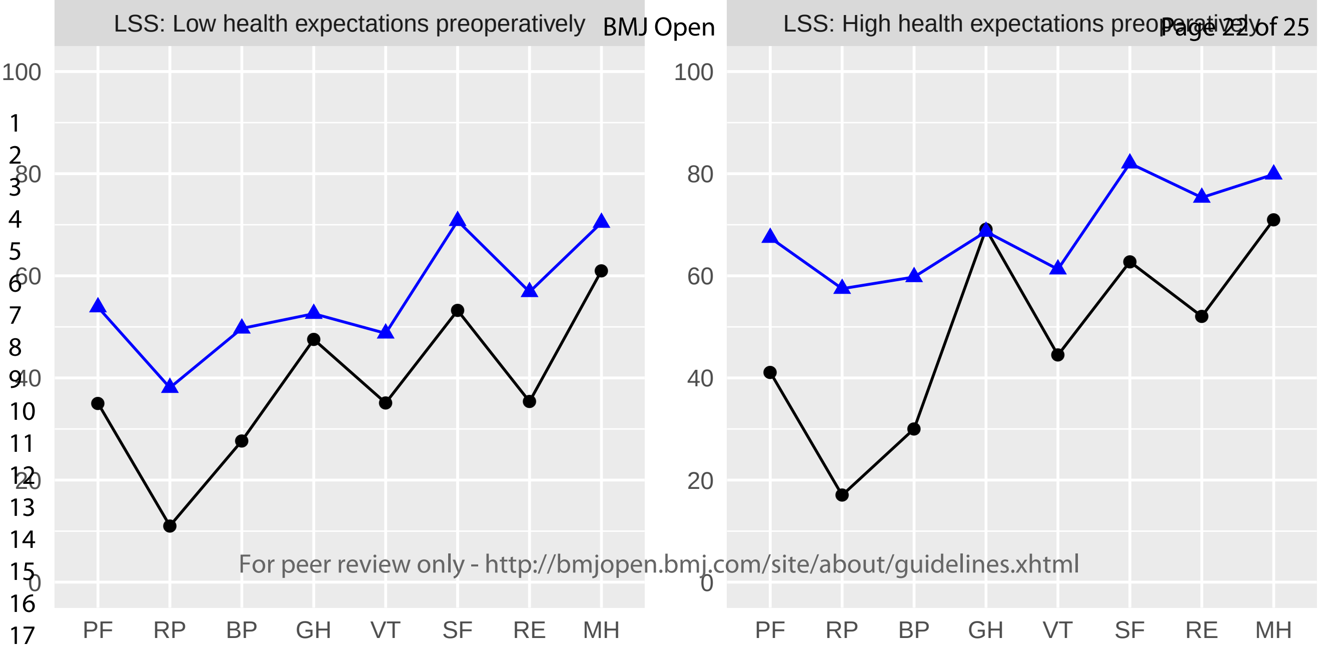

For peer review only - http://bmjopen.bmj.com/site/about/guidelines.xhtml

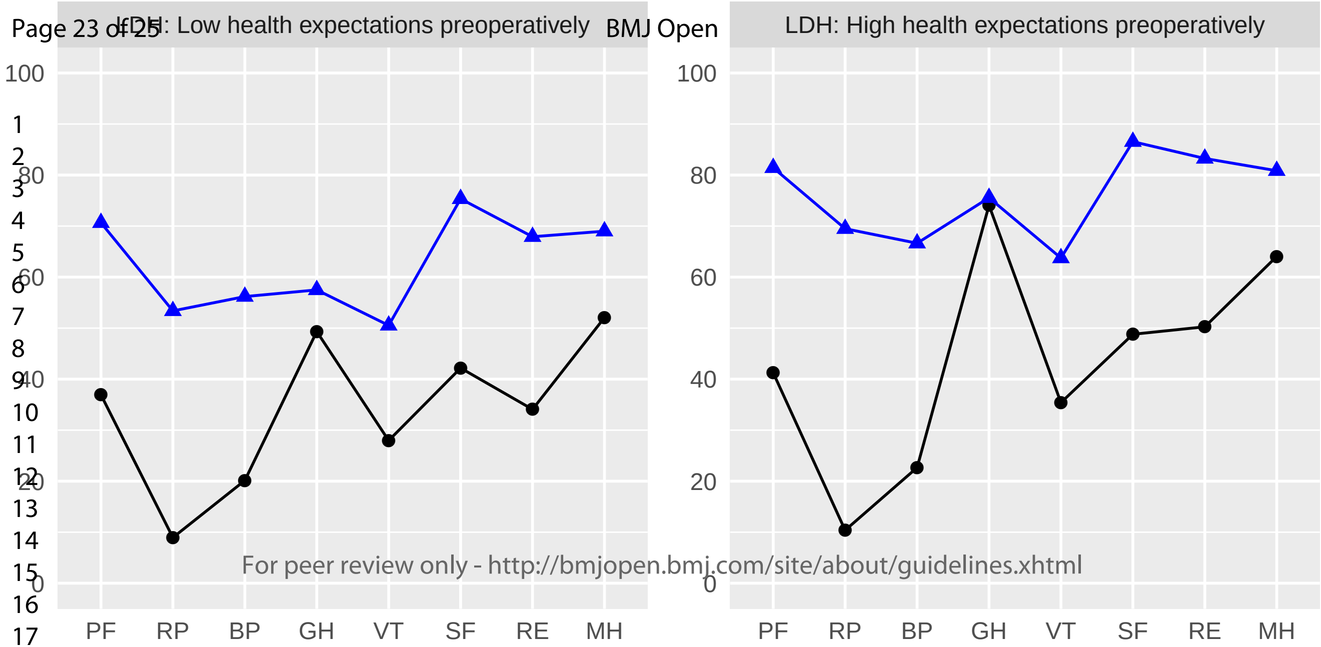
LDH: Low health expectations preoperatively
LDH: High health expectations preoperatively
100
80
60
40
20
0
PF
RP
BP
GH
VT
SF
RE
MH

Table S1 Comparison of baseline characteristics between included and excluded patients.

	LSS		LDH	
	Included	*Excluded*	*Included*	*Excluded*
n	3969	5639	5960	8559
Age, mean (SD)	66 (10.1)	67.8 (10.8)	44.8 (12.6)	44.4 (13.1)
BMI, mean (SD)	27.6 (4.01)	27.7 (4.18)	26.3 (4.12)	26.6 (4.28)
Women, n (%)	1819 (45.8)	2855 (50.6)	2635 (44.2)	3760 (43.9)

Table S2 Distribution of the SF-36 item 11c (I expect my health to get worse) responses preoperatively and year 1 for LSS (n=3969) and LDH (n=5960).

		1. Definitely True	2. Mostly True	3. Don't know	4. Mostly False	5. Definitely False
LSS, n (%)	*Preop*	78 (1.97)	226 (5.69)	1197 (30.2)	935 (23.6)	1533 (38.6)
	Year 1	157 (3.96)	489 (12.3)	1471 (37.1)	724 (18.2)	1128 (28.4)
LDH, n (%)	*Preop*	93 (1.56)	206 (3.46)	1034 (17.3)	1307 (21.9)	3320 (55.7)
	Year 1	137 (2.3)	427 (7.16)	1483 (24.9)	1270 (21.3)	2643 (44.3)

Table S3 Preoperative and year one postoperative SF-36 data for patients treated surgically for one-level central spinal stenosis between 2007 to 2016.

	Low future health expectations preoperatively			High future health expectations preoperatively		
	Preop Mean (95% CI)	*Year one Mean (95% CI)*	*SRM (95% CI)*	*Preop Mean (95% CI)*	*Year one Mean (95% CI)*	*SRM (95% CI)*
**PF**	35 (34;36)	54 (53;55)	0.78 (0.72;0.83)	41 (40;42)	67 (66;68)	1.06 (1.01;1.11)
**RP**	11 (9.7;12)	38 (36;40)	0.62 (0.57;0.67)	17 (16;18)	57 (56;59)	0.87 (0.82;0.92)
**BP**	28 (27;28)	50 (49;51)	0.86 (0.82;0.91)	30 (29;31)	60 (59;61)	1.08 (1.04;1.12)
**GH**	48 (47;48)	53 (51;54)	0.27 (0.22;0.32)	69 (69;70)	69 (68;69)	-0.024 (-0.063;0.015)
**VT**	35 (34;36)	49 (47;50)	0.59 (0.54;0.64)	44 (44;45)	61 (60;62)	0.68 (0.64;0.72)
**SF**	53 (52;54)	71 (69;72)	0.61 (0.56;0.67)	63 (62;64)	82 (81;83)	0.66 (0.62;0.71)
**RE**	35 (33;37)	57 (55;59)	0.42 (0.37;0.47)	52 (50;54)	75 (74;77)	0.46 (0.42;0.5)
**MH**	61 (60;62)	70 (69;71)	0.45 (0.4;0.5)	71 (70;72)	80 (79;81)	0.44 (0.4;0.48)

Table S4 Preoperative and year one postoperative SF-36 data for patients treated surgically for one-level disk herniation between 2007 to 2016.

	Low future health expectations preoperatively			High future health expectations preoperatively		
	Preop Mean (95% CI)	*Year one Mean (95% CI)*	*SRM (95% CI)*	*Preop Mean (95% CI)*	*Year one Mean (95% CI)*	*SRM (95% CI)*
**PF**	37 (36;38)	71 (69;72)	1.2 (1.1;1.3)	41 (41;42)	81 (81;82)	1.54 (1.49;1.58)
**RP**	8.9 (7.8;10)	53 (51;56)	0.99 (0.93;1.1)	10 (9.7;11)	69 (68;71)	1.37 (1.32;1.41)
**BP**	20 (19;21)	56 (55;58)	1.3 (1.2;1.3)	23 (22;23)	67 (66;67)	1.55 (1.51;1.59)
**GH**	49 (48;50)	57 (56;59)	0.39 (0.34;0.45)	74 (74;75)	76 (75;76)	0.076 (0.048;0.11)
**VT**	28 (27;29)	51 (49;52)	0.88 (0.81;0.94)	35 (35;36)	64 (63;64)	1.1 (1.1;1.1)
**SF**	42 (41;44)	75 (74;77)	1 (0.97;1.1)	49 (48;50)	87 (86;87)	1.2 (1.1;1.2)
**RE**	34 (32;36)	68 (66;70)	0.67 (0.61;0.73)	50 (49;52)	83 (82;84)	0.66 (0.62;0.69)
**MH**	52 (51;53)	69 (68;70)	0.74 (0.68;0.79)	64 (63;65)	81 (80;81)	0.79 (0.75;0.82)

For peer review only

STROBE 2007 (v4) Statement—Checklist of items that should be included in reports of *cohort studies*

Section/Topic	Item #	Recommendation	Reported on page #
**Title and abstract**	1	(*a*) Indicate the study's design with a commonly used term in the title or the abstract	2
		(*b*) Provide in the abstract an informative and balanced summary of what was done and what was found	2
**Introduction**			
Background/rationale	2	Explain the scientific background and rationale for the investigation being reported	4
Objectives	3	State specific objectives, including any prespecified hypotheses	4,5
**Methods**			
Study design	4	Present key elements of study design early in the paper	5
Setting	5	Describe the setting, locations, and relevant dates, including periods of recruitment, exposure, follow-up, and data collection	5
Participants	6	(*a*) Give the eligibility criteria, and the sources and methods of selection of participants. Describe methods of follow-up	5
		(*b*) For matched studies, give matching criteria and number of exposed and unexposed	NA
Variables	7	Clearly define all outcomes, exposures, predictors, potential confounders, and effect modifiers. Give diagnostic criteria, if applicable	5,6
Data sources/ measurement	8*	For each variable of interest, give sources of data and details of methods of assessment (measurement). Describe comparability of assessment methods if there is more than one group	5
Bias	9	Describe any efforts to address potential sources of bias	NA
Study size	10	Explain how the study size was arrived at	5,7
Quantitative variables	11	Explain how quantitative variables were handled in the analyses. If applicable, describe which groupings were chosen and why	7
Statistical methods	12	(*a*) Describe all statistical methods, including those used to control for confounding	7
		(*b*) Describe any methods used to examine subgroups and interactions	NA
		(*c*) Explain how missing data were addressed	7
		(*d*) If applicable, explain how loss to follow-up was addressed	NA
		(*e*) Describe any sensitivity analyses	NA
**Results**			

For peer review only

Participants	13*	(a) Report numbers of individuals at each stage of study—eg numbers potentially eligible, examined for eligibility, confirmed eligible, included in the study, completing follow-up, and analysed	7
		(b) Give reasons for non-participation at each stage	NA
		(c) Consider use of a flow diagram	NA
Descriptive data	14*	(a) Give characteristics of study participants (eg demographic, clinical, social) and information on exposures and potential confounders	7, Table S1
		(b) Indicate number of participants with missing data for each variable of interest	7
		(c) Summarise follow-up time (eg, average and total amount)	7
Outcome data	15*	Report numbers of outcome events or summary measures over time	NA
Main results	16	(*a*) Give unadjusted estimates and, if applicable, confounder-adjusted estimates and their precision (eg, 95% confidence interval). Make clear which confounders were adjusted for and why they were included	7-9, Tables 3-4
		(*b*) Report category boundaries when continuous variables were categorized	NA
		(*c*) If relevant, consider translating estimates of relative risk into absolute risk for a meaningful time period	NA
Other analyses	17	Report other analyses done—eg analyses of subgroups and interactions, and sensitivity analyses	NA
**Discussion**			
Key results	18	Summarise key results with reference to study objectives	9
**Limitations**			
Interpretation	20	Give a cautious overall interpretation of results considering objectives, limitations, multiplicity of analyses, results from similar studies, and other relevant evidence	9-12
Generalisability	21	Discuss the generalisability (external validity) of the study results	9-12
**Other information**			
Funding	22	Give the source of funding and the role of the funders for the present study and, if applicable, for the original study on which the present article is based	13

*Give information separately for cases and controls in case-control studies and, if applicable, for exposed and unexposed groups in cohort and cross-sectional studies.

Note: An Explanation and Elaboration article discusses each checklist item and gives methodological background and published examples of transparent reporting. The STROBE checklist is best used in conjunction with this article (freely available on the Web sites of PLoS Medicine at http://www.plosmedicine.org/, Annals of Internal Medicine at http://www.annals.org/, and Epidemiology at http://www.epidem.com/). Information on the STROBE Initiative is available at www.strobe-statement.org.

For peer review only

Associations between future health expectations and patient satisfaction after lumbar spine surgery: A longitudinal observational study of 9929 lumbar spine surgery procedures

Journal:	*BMJ Open*
Manuscript ID	bmjopen-2023-074072.R1
Article Type:	Original research
Date Submitted by the Author:	21-Jun-2023
Complete List of Authors:	Joelson, Anders; Orebro universitet<br>Szigethy, Lilla; Region Orebro lan<br>Wildeman, Peter; Orebro universitet<br>Sigmundsson, Freyr Gauti; Orebro universitet<br>Karlsson, Jan; Orebro universitet
**Primary Subject Heading**:	Surgery
Secondary Subject Heading:	Patient-centred medicine
Keywords:	Spine < ORTHOPAEDIC & TRAUMA SURGERY, Adult orthopaedics < ORTHOPAEDIC & TRAUMA SURGERY, Quality of Life

SCHOLARONE™
Manuscripts

I, the Submitting Author has the right to grant and does grant on behalf of all authors of the Work (as defined in the below author licence), an exclusive licence and/or a non-exclusive licence for contributions from authors who are: i) UK Crown employees; ii) where BMJ has agreed a CC-BY licence shall apply, and/or iii) in accordance with the terms applicable for US Federal Government officers or employees acting as part of their official duties; on a worldwide, perpetual, irrevocable, royalty-free basis to BMJ Publishing Group Ltd ("BMJ") its licensees and where the relevant Journal is co-owned by BMJ to the co-owners of the Journal, to publish the Work in this journal and any other BMJ products and to exploit all rights, as set out in our licence.

The Submitting Author accepts and understands that any supply made under these terms is made by BMJ to the Submitting Author unless you are acting as an employee on behalf of your employer or a postgraduate student of an affiliated institution which is paying any applicable article publishing charge ("APC") for Open Access articles. Where the Submitting Author wishes to make the Work available on an Open Access basis (and intends to pay the relevant APC), the terms of reuse of such Open Access shall be governed by a Creative Commons licence – details of these licences and which Creative Commons licence will apply to this Work are set out in our licence referred to above.

Other than as permitted in any relevant BMJ Author's Self Archiving Policies, I confirm this Work has not been accepted for publication elsewhere, is not being considered for publication elsewhere and does not duplicate material already published. I confirm all authors consent to publication of this Work and authorise the granting of this licence.

For peer review only

Associations between future health expectations and patient satisfaction after lumbar spine surgery: A longitudinal observational study of 9929 lumbar spine surgery procedures

Anders Joelson, MD, PhD[1,2], Lilla Szigethy, MD[2], Peter Wildeman, MD, PhD[1,2], Freyr Gauti Sigmundsson, MD, PhD[1,2], Jan Karlsson, PhD[3]

1. Örebro University School of Medical Sciences, SE-70182 Örebro, Sweden
2. Department of Orthopaedics, Örebro University Hospital, SE-70185 Örebro, Sweden
3. University Health Care Research Center, Faculty of Medicine and Health, Örebro University, SE-70182, Örebro, Sweden

Correspondence:

Anders Joelson

E-mail address: anders.joelson@oru.se

Tel: +46196021000 Fax: +46196025337

For peer review only

Abstract

Objective: This study aimed to investigate the associations between general health expectations and patient satisfaction with treatment for the two common spine surgery procedures diskectomy for lumbar disk herniations (LDH) and decompression for lumbar spinal stenosis (LSS).

Design: Register study with prospectively collected preoperative and one-year postoperative data.

Setting: National outcome data from Swespine, the National Swedish spine register.

Participants: A total of 9929 patients, aged between 20 and 85 years, who were self-reported non-smokers, and were operated between 2007 and 2016 for one-level LSS without degenerative spondylolisthesis, or one-level LDH, were identified in the national Swedish spine register (Swespine). We used SF-36 items 11c and 11d to assess future health expectations and present health perceptions. Satisfaction with treatment was assessed using the Swespine satisfaction item.

Interventions: One-level diskectomy for LDH or one-level decompression for LSS.

Primary outcome measures: Satisfaction with treatment.

Results: For LSS, the year one satisfaction ratio amongst patients with negative future health expectations preoperatively was 60% (95% CI 58 to 63), while it was 75% (95% CI 73 to 76) for patients with positive future health expectations preoperatively. The corresponding numbers for LDH were 73% (95% CI 71 to 75) and 84% (95% CI 83 to 85) respectively.

Conclusions: Patients operated for the common lumbar spine diseases LSS or LDH, with negative future general health expectations, were significantly less satisfied with treatment than patients with positive expectations with regards to future general health. These findings are important for patients, and for the surgeons who counsel them, when surgery is a treatment option for LSS or LDH.

Strengths and limitations

1. The study includes a large number of patients from a national database with high, stable coverage.

2. We recognize the inherent limitations of register data, such as lack of confounder information, missing data, or unknown data quality.

3. The data were incomplete in 58% of the procedures which is a major limitation of our study that affects the internal and external validity of our findings.

For peer review only

Introduction

Lumbar degenerative spine diseases are major causes of pain and disability worldwide [1, 2]. Patient-reported outcome measures (PROMs) and patient satisfaction scales are commonly used to evaluate treatment outcomes after lumbar spine surgery [3]. However, there are inconsistencies between PROM changes and treatment satisfaction when evaluating surgical outcome. For example, Chotai et al. [4] found that 83% of patients were satisfied with treatment after elective surgery for degenerative spine disease, whereas only 62% achieved minimal important change (MIC) for the Oswestry/neck disability indices. Furthermore, Godil et al. [5], in an analysis using receiver operating characteristics (ROC) curves, found that improvement in the Oswestry disability index failed to discriminate between satisfaction and dissatisfaction with treatment with good accuracy after spine surgery. In contrast, Copay et al. [6] found a strong association between the Oswestry disability index and patient satisfaction after lumbar surgery. The variety of results suggest that patient satisfaction is also influenced by factors other than PROM changes, such as expectations, socioeconomic factors, and mental health.

The impact of psychological factors on the outcomes of spine surgery has been thoroughly researched [7, 8, 9, 10]. In addition, Iderberg et al. [11] demonstrated that socioeconomic indicators are associated with the outcomes of surgery for lumbar spinal stenosis. Moreover, several reports have shown that preoperative expectations on recovery predict the outcome of spine surgery [12, 13, 14, 15, 16, 17, 18, 19]. Interestingly, previous studies have shown that expectations on future general health are associated with mortality and functional decline [20, 21, 22]. These findings raise the question of whether there is also an association between future general health expectations and the outcome of health interventions like spine surgery. However,

For peer review only

data on the expectations on future general health expectations and outcomes of spine surgery are limited. Therefore, the current study aimed to investigate the associations between future general health expectations and patient satisfaction following surgery for degenerative spine diseases.

Methods

Study design

The present study was a register study, with prospectively collected longitudinal data from Swespine, the national Swedish spine register [23].

The national Swedish spine register (Swespine)

The Swespine register was launched in 1992 and covers 90% of the spine units in Sweden. The one-year follow-up rate is 70-75% [23]. The register includes data on diagnoses, surgical procedures, complications, and patient-reported outcome measures. The surgeon is responsible for submitting data about the surgery.

Patient data set

Patients, aged between 20 and 85 years, who were self-reported non-smokers, and were surgically treated between 2007 and 2016 for one-level lumbar spinal stenosis (LSS) without degenerative spondylolisthesis, or one-level lumbar disk herniation (LDH), were identified in Swespine.

For peer review only

Measures

The SF-36 is an 8-dimensional, 36-item, self-administered HRQoL instrument for the assessment of general HRQoL [24]. The instrument has 6 items for assessment of general health perceptions: item 1 (present health), item 2 (health transition), item 11a (health comparison), item 11b (health context), item 11c (future health), and item 11d (present health). Items 1 and 11a-d form the general health domain of SF-36. In our study, we used items 11c and 11d to assess future health expectations and present health perceptions (table 1). We grouped future health expectations into negative (pessimistic) health expectations (item 11c response options 1, 2, and 3) and positive (optimistic) health expectations (item 11c response options 4 and 5). We grouped present health perceptions into positive present health perceptions (item 11d response options 1, 2, and 3) and negative present health perceptions (item 11c response options 4 and 5). We used the Swedish translation of SF-36 version 1 in our study [25].

Satisfaction with treatment was assessed using the Swespine satisfaction item (table 1). In our analysis, we grouped satisfaction with treatment into satisfied (response option 1) and dissatisfied (response options 2 and 3).

For peer review only

Table 1 Questions and response options.

	**Question**	**Response options**
Future health (SF-36 Item 11c)	I expect my health to get worse.	1. Definitely True<br>2. Mostly True<br>3. Don't Know<br>4. Mostly False<br>5. Definitely False
Present health (SF-36 Item 11d)	My health is excellent.	1. Definitely True<br>2. Mostly True<br>3. Don't Know<br>4. Mostly False<br>5. Definitely False
Satisfaction (Swespine)	What is your attitude regarding the outcome of your spine surgery?	1. I am satisfied<br>2. I am uncertain<br>3. I am dissatisfied

Statistics

Data are presented as mean and standard deviation (SD) and/or 95% confidence intervals (CIs). Bootstrapping was used to calculate the CIs [26]. Standardized response mean (SRM) for paired data was used to evaluate effect size [27]. The SRM was interpreted as follows: <0.2 no effect, 0.2 to 0.4 small effect, 0.5 to 0.7 moderate effect, >0.7 large effect [28]. Multiple linear logistic regression analysis was used to model the relationship between the outcome and covariates [29]. All covariates of the model were binary, continuous covariates were dichotomised by using their respective median values.

Patient and public involvement

The patients and the public were not involved in the design, recruitment, conduct, or dissemination plans of this research.

For peer review only

Results

A total of 24127 surgical procedures for the treatment of the lumbar spine diseases LDH and LSS were included in Swespine between 2007 and 2016. Preoperative or one-year postoperative SF-36 data were incomplete for 14198 (58%) of the procedures which provided 9929 procedures eligible for analysis. The baseline characteristics of the included and excluded patients are presented in supplementary table S1.

For LSS, 1501 (38%) of 3969 patients had negative future health expectations preoperatively and 2117 (53%) of 3969 patients had negative future health expectations at the year one follow-up. For LDH, the corresponding number was 1333 (22%) of 5960 patients preoperatively and 2047 (34%) of 5960 patients at the year one follow-up (supplementary table S2).

The preoperative characteristics of the patients with negative and positive future health expectations are presented in table 2. The SF-36 profiles preoperatively and one year postoperatively are shown in supplementary figures S1 and S2 and the effect sizes of changes are shown in supplementary tables S3 and S4. For LSS, the satisfaction ratio year one postoperatively amongst patients with negative future health expectations preoperatively was 60%, while it was 75% for patients with positive future health expectations (table 3). The corresponding levels for LDH were 73% and 84% respectively. The differences in satisfaction ratios were statistically significant (non-overlapping CIs). Table 4 summarises multiple linear logistic regression models for patient satisfaction one year postoperatively using preoperative future health expectations, preoperative present health perceptions, age, gender, and BMI as covariates.

For peer review only

Table 2 Comparison of characteristics between patients with positive and negative future health expectations preoperatively.

	LSS (n=3969)		LDH (n=5960)	
	Negative future health expectations	*Positive future health expectations*	*Negative future health expectations*	*Positive future health expectations*
n (%)	1501 (37.8)	2468 (62.2)	1333 (22.4)	4627 (77.6)
Age, mean (SD)	67.8 (10)	64.9 (10.1)	46.8 (13.8)	44.3 (12.1)
BMI, mean (SD)	27.7 (4.0)	27.5 (4.0)	26.7 (4.4)	26.1 (4.0)
Women, n (%)	709 (47.2)	1110 (45)	596 (44.7)	2039 (44.1)

Table 3 Patient satisfaction one year postoperatively for LSS (n=3969) and LDH (n=5960).

	Negative future health expectations preoperatively	Positive future health expectations preoperatively
LSS, % satisfied (95% CI) (n/total)	60.4 (57.8;62.8) (906/1501)	74.5 (72.9;76.1) (1839/2468)
LDH, % satisfied (95% CI) (n/total)	73 (70.7;75.2) (973/1333)	83.8 (82.8;84.9) (3879/4627)

Table 4 Multiple linear logistic regression models for patient satisfaction one year postoperatively for LSS (n=3969) and LDH (n=5960). Hosmer-Lemeshow goodness-of-fit test: LSS p=0.52, LDH p=0.48.

	LSS	LDH
Intercept, OR (95% CI)	0.981 (0.834;1.15)	1.9 (1.6;2.25)
Future health, OR (95% CI)	1.71 (1.48;1.97)	1.72 (1.48;1.99)
Present health, OR (95% CI)	1.42 (1.23;1.64)	1.47 (1.28;1.69)
Age, OR (95% CI)	1.38 (1.2;1.59)	1.14 (0.994;1.3)
Gender, OR (95% CI)	1.13 (0.98;1.29)	1.17 (1.02;1.33)
BMI, OR (95% CI)	1.25 (1.09;1.44)	1.14 (0.998;1.3)

Discussion

In this paper, we found that patients, operated for the common lumbar spine diseases LSS or LDH, with negative future general health expectations preoperatively, were significantly less satisfied with treatment compared with patients with positive expectations with regards to future

For peer review only

general health. To our knowledge, our study is the first to report data on the association between expectations on future general health assessed preoperatively and patient satisfaction after lumbar spine surgery. Clinicians that are using SF-36 in the preoperative evaluation of patients scheduled for LSS or LDH surgery will get additional information by analysing the answer to item 11c. Assessment of item 11c might be useful and valuable in practice settings in the identification of patients that might benefit from a more active rehabilitation or follow-up.

Belayneh et al. [19] studied the impact of future health expectations on the outcome after surgical repair of proximal humeral fractures and found that patients with positive expectations on their health, early following the injury, had better long-term outcomes. The authors evaluated future health expectations using a question with exactly the same wording as used in our study. This strengthens the assumption that SF-36 item 11c may be used to assess future health expectations in the field of orthopedic surgery. We agree with the authors that health care providers should communicate with patients, to ensure that they are setting clear expectations of the benefits and risks for each patient.

Iversen et al. [12] studied several expectations summed across the domains pain reduction, physical functioning, and social functioning to evaluate the prognostic importance of preoperative expectations on the treatment outcomes of LSS surgery and found that patients' expectations influence recovery from surgery at six months. The authors concluded that clinicians should discuss expectations with patients preoperatively in order to establish realistic goals and to enable patients to actively engage in their rehabilitation, a conclusion that we agree with.

For peer review only

Standard surgical procedures for the treatment of LDH and LSS are considered safe and beneficial treatment options [30, 31]. However, there are rare but serious complications such as nerve rot lesions [31]. As the main goal of elective surgery for LDH and LSS is to improve patient quality of life, it is important to weigh the benefits against the potential risks when discussing treatment options with patients. For patients with negative future health expectations, our data suggests that the satisfaction rate for LSS surgery could be as low as 60%. This information is important from a shared decision-making perspective when balancing the benefits and risks of surgery.

We used SF-36 item 11c to assess future health expectations. Previous studies have indicated that the wording of item 11c sometimes is seen to be unnecessarily negative [32]. Furthermore, Shaples et al. [33] speculated that elderly people might be reluctant to consider questions about worsening in health but concluded that the item did not affect the internal consistency of the SF-36 general health (GH) domain. Although there are some concerns about the design of item 11c, we do not expect that these concerns would invalidate the use of item 11c for the assessment of future health expectations.

Several factors may affect future health expectations. The SF-36 health profiles presented in supplementary figures S1 and S2, and supplementary tables S3 and S4 indicate that negative future health expectations do not only affect the GH domain, as the patients report lower scores on all SF-36 domains. This illustrates that negative future health expectations affects several dimensions of HRQoL.

For peer review only

The five-factor model is commonly used in psychology to model different personality traits [34]. The model uses five orthogonal trait dimensions to describe different personalities: neuroticism (N), extraversion (E), openness (O), agreeableness (A), and conscientiousness (C). Chapman et al. [35] studied the influence of the five-factor model personality traits on perceived health using the NEO Five-Factor Inventory (NEO-FFI) [36] and SF-36. Low N scores and high E scores were associated with positive future health expectations. However, although the differences in the personality trait scores were statistically significant, the actual differences were small. Hendriks et al. [37] found that patient satisfaction was only marginally associated with personality. Consequently, the influence of different personality traits on health expectations and patient satisfaction remains unclear, and this field may benefit from further research.

Notably, 38% of the patients with LSS had negative future health expectations preoperatively whereas 53% had negative future health expectations at the year one follow-up. The corresponding levels for LDH were 22% and 34% respectively. One possible explanation is that preoperatively the patients expect an improvement in health because of the forthcoming operation, while at one year after the operation, the patients may be more neutral or pessimistic about future health improvements. This indicates that questions about future health expectations must be interpreted with caution when asked before and after a health intervention.

The results of our multiple linear logistic regression analysis indicated that there was an association between general health assessments (present and future) and patient satisfaction after surgery for LDH and LSS. Ferrato et al. [21] reported that there are indications that queries about future health expectations are more useful than those about past health changes in mortality predictions. However, our study could not confirm that future health expectations had larger

For peer review only - http://bmjopen.bmj.com/site/about/guidelines.xhtml

impact on patient satisfaction than present health perceptions since the odds ratios for present and future health had overlapping CIs. Age, gender, and BMI made only minor contributions as predictors of patient satisfaction one year after LDH surgery, whereas age had some impact on satisfaction after surgery for LSS.

Our findings should be evaluated in the light of several limitations. First, we recognize the inherent limitations of register data, such as lack of confounder information, missing data, or unknown data quality [38]. Second, information on co-morbidities that might affect patient satisfaction was lacking. Third, the data were incomplete in 58% of the procedures**.** This is a major limitation of our study that affects the internal and external validity of our findings. Fourth, data on socioeconomic factors were lacking. The study of Iderberg et al. [11] demonstrated that socioeconomic indicators were associated with outcomes of surgery for LSS.

Conclusions

Patients surgically treated for the common lumbar spine diseases LSS or LDH, with negative future general health expectations, were significantly less satisfied with treatment compared with patients with positive expectations on future general health. The findings of this study can be used in the shared decision-making process when surgery is a treatment option for patients with LSS or LDH to establish realistic expectations and to enable patients to actively engage in rehabilitation.

For peer review only

Contributions

All authors designed the study. AJ analyzed the data. All authors interpreted the data. AJ wrote the manuscript with contributions from LS, PW, FGS, and JK. All authors approved the final version of the manuscript.

Funding

This research received no specific grant from any funding agency in the public, commercial, or not-for-profit sectors.

Competing interests

None.

Patient consent

Not applicable.

Ethics approval

The study was approved by the Swedish Ethical Review Authority (registration number: 2020-03557).

Data availability statement

Data may be obtained from a third party and are not publicly available. Data are available from the national Swedish spine register (Swespine) after approval by the Swedish Ethical Review Authority and according to the regulations in the General Data Protection Regulation and the Swedish Patient Data Act.

For peer review only

References

1. Ravindra VM, Senglaub SS, Rattani A, Dewan MC, Härtl R, Bisson E, Park KB, Shrime MG. Degenerative Lumbar Spine Disease: Estimating Global Incidence and Worldwide Volume. Global Spine J. 2018 Dec;8(8):784-794.

2. Hoy D, March L, Brooks P, Blyth F, Woolf A, Bain C, Williams G, Smith E, Vos T, Barendregt J, Murray C, Burstein R, Buchbinder R. The global burden of low back pain: estimates from the Global Burden of Disease 2010 study. Ann Rheum Dis. 2014 Jun;73(6):968-74.

3. Clement RC, Welander A, Stowell C, Cha TD, Chen JL, Davies M, Fairbank JC, Foley KT, Gehrchen M, Hagg O, Jacobs WC, Kahler R, Khan SN, Lieberman IH, Morisson B, Ohnmeiss DD, Peul WC, Shonnard NH, Smuck MW, Solberg TK, Stromqvist BH, Hooff MLV, Wasan AD, Willems PC, Yeo W, Fritzell P. A proposed set of metrics for standardized outcome reporting in the management of low back pain. Acta Orthop. 2015;86(5):523-33.

4. Chotai S, Sivaganesan A, Parker SL, McGirt MJ, Devin CJ. Patient-Specific Factors Associated With Dissatisfaction After Elective Surgery for Degenerative Spine Diseases. Neurosurgery. 2015 Aug;77(2):157-63; discussion 163.

5. Godil SS, Parker SL, Zuckerman SL, Mendenhall SK, Devin CJ, Asher AL, McGirt MJ. Determining the quality and effectiveness of surgical spine care: patient satisfaction is not a valid proxy. Spine J. 2013 Sep;13(9):1006-12.

For peer review only

6. Copay AG, Martin MM, Subach BR, Carreon LY, Glassman SD, Schuler TC, Berven S. Assessment of spine surgery outcomes: inconsistency of change amongst outcome measurements. Spine J. 2010 Apr;10(4):291-6.

7. Sinikallio S, Aalto T, Koivumaa-Honkanen H, Airaksinen O, Herno A, Kröger H, Viinamäki H. Life dissatisfaction is associated with a poorer surgery outcome and depression among lumbar spinal stenosis patients: a 2-year prospective study. Eur Spine J. 2009 Aug;18(8):1187-93.

8. Sinikallio S, Aalto T, Airaksinen O, Lehto SM, Kröger H, Viinamäki H. Depression is associated with a poorer outcome of lumbar spinal stenosis surgery: a two-year prospective follow-up study. Spine (Phila Pa 1976). 2011 Apr;36(8):677-82.

9. Pakarinen M, Vanhanen S, Sinikallio S, Aalto T, Lehto SM, Airaksinen O, Viinamäki H. Depressive burden is associated with a poorer surgical outcome among lumbar spinal stenosis patients: a 5-year follow-up study. Spine J. 2014 Oct;14(10):2392-6.

10. Miller JA, Derakhshan A, Lubelski D, Alvin MD, McGirt MJ, Benzel EC, Mroz TE. The impact of preoperative depression on quality of life outcomes after lumbar surgery. Spine J. 2015 Jan;15(1):58-64.

11. Iderberg H, Willers C, Borgström F, Hedlund R, Hägg O, Möller H, Ornstein E, Sandén B, Stalberg H, Torevall-Larsson H, Tullberg T, Fritzell P. Predicting clinical outcome and length of

For peer review only

sick leave after surgery for lumbar spinal stenosis in Sweden: a multi-register evaluation. Eur Spine J. 2019 06;28(6):1423-1432.

12. Iversen MD, Daltroy LH, Fossel AH, Katz JN. The prognostic importance of patient pre-operative expectations of surgery for lumbar spinal stenosis. Patient Educ Couns. 1998 Jun;34(2):169-78.

13. de Groot KI, Boeke S, Passchier J. Preoperative expectations of pain and recovery in relation to postoperative disappointment in patients undergoing lumbar surgery. Med Care. 1999 Feb;37(2):149-56.

14. Lutz GK, Butzlaff ME, Atlas SJ, Keller RB, Singer DE, Deyo RA. The relation between expectations and outcomes in surgery for sciatica. J Gen Intern Med. 1999 Dec;14(12):740-4.

15. Toyone T, Tanaka T, Kato D, Kaneyama R, Otsuka M. Patients' expectations and satisfaction in lumbar spine surgery. Spine (Phila Pa 1976). 2005 Dec;30(23):2689-94.

16. Rönnberg K, Lind B, Zoëga B, Halldin K, Gellerstedt M, Brisby H. Patients' satisfaction with provided care/information and expectations on clinical outcome after lumbar disc herniation surgery. Spine (Phila Pa 1976). 2007 Jan;32(2):256-61.

17. Yee A, Adjei N, Do J, Ford M, Finkelstein J. Do patient expectations of spinal surgery relate to functional outcome? Clin Orthop Relat Res. 2008 May;466(5):1154-61.

For peer review only

18. Mannion AF, Junge A, Elfering A, Dvorak J, Porchet F, Grob D. Great expectations: really the novel predictor of outcome after spinal surgery? Spine (Phila Pa 1976). 2009 Jul;34(15):1590-9.

19. Belayneh R, Lott A, Haglin J, Zuckerman J, Egol K. The role of patients' overall expectations of health on outcomes following proximal humerus fracture repair. Orthop Traumatol Surg Res. 2021 Dec;107(8):103043.

20. Wang C, Satariano WA. Self-rated current and future health independently predict subsequent mortality in an aging population. J Gerontol A Biol Sci Med Sci. 2007 Dec;62(12):1428-34.

21. Ferraro KF, Wilkinson LR. Alternative Measures of Self-Rated Health for Predicting Mortality Among Older People: Is Past or Future Orientation More Important? Gerontologist. 2015 Oct;55(5):836-44.

22. Lee Y. The predictive value of self assessed general, physical, and mental health on functional decline and mortality in older adults. J Epidemiol Community Health. 2000 Feb;54(2):123-9.

23. Strömqvist B, Fritzell P, Hägg O, Jönsson B, Sandén B, Swedish Society of Spinal Surgeons. Swespine: The Swedish spine register: The 2012 report. Eur Spine J. 2013 Apr;22(4):953-74.

24. Ware JE Jr, Sherbourne CD. The MOS 36-item short-form health survey (SF-36). I. Conceptual framework and item selection. Med Care. 1992 Jun;30(6):473-83.

For peer review only

25. Sullivan M, Karlsson J, Ware JE Jr. The Swedish SF-36 health survey-I. Evaluation of data quality, scaling assumptions, reliability and construct validity across general populations in Sweden. Soc Sci Med. 1995;41(10):1349-58.

26. Bland JM, Altman DG. Statistics Notes: Bootstrap resampling methods. BMJ. 2015 Jun;350:h2622.

27. Fayers PM, Machin D. Quality of life: The assessment, analysis and reporting of patient-reported outcomes. 3rd ed. Chichester: John Wiley and Sons Ltd; 2016.

28. Cohen J. Statistical power analysis for the behavioral sciences. 2nd ed. Hillsdale, NJ: Lawrence Erlbaum Associates; 1988.

29. Altman DG. Practical statistics for medical research. London: Chapman and Hall; 1991.

30. Weinstein JN, Tosteson TD, Lurie JD, Tosteson ANA, Blood E, Hanscom B, Herkowitz H, Cammisa F, Albert T, Boden SD, Hilibrand A, Goldberg H, Berven S, An H, SPORT Investigators. Surgical versus nonsurgical therapy for lumbar spinal stenosis. N Engl J Med. 2008 Feb;358(8):794-810.

31. Weinstein JN, Lurie JD, Tosteson TD, Tosteson ANA, Blood EA, Abdu WA, Herkowitz H, Hilibrand A, Albert T, Fischgrund J. Surgical versus nonoperative treatment for lumbar disc

For peer review only

herniation: four-year results for the Spine Patient Outcomes Research Trial (SPORT). Spine (Phila Pa 1976). 2008 Dec;33(25):2789-800.

32. Hayes V, Morris J, Wolfe C, Morgan M. The SF-36 health survey questionnaire: is it suitable for use with older adults? Age Ageing. 1995 Mar;24(2):120-5.

33. Sharples LD, Todd CJ, Caine N, Tait S. Measurement properties of the Nottingham health profile and short form 36 health status measures in a population sample of elderly people living at home: Results from ELPHS. Br J Health Psychol. 2000;5:217-233.

34. Goldberg LR. The structure of phenotypic personality traits. Am Psychol. 1993 Jan;48(1):26-34.

35. Chapman BP, Duberstein PR, Sörensen S, Lyness JM. Personality and perceived health in older adults: the five factor model in primary care. J Gerontol B Psychol Sci Soc Sci. 2006 Nov;61(6):P362-5.

36. McCrae RR, Costa PT Jr. A contemplated revision of the NEO Five-Factor Inventory. Pers Individ Dif. 2004 FEB;36(3):587-596.

37. Hendriks A, Smets E, Vrielink M, Van Es S, De Haes J. Is personality a determinant of patient satisfaction with hospital care? int j qual health care. 2006 APR;18(2):152-158.

For peer review only

38. Thygesen LC, Ersbøll AK. When the entire population is the sample: strengths and limitations in register-based epidemiology. Eur J Epidemiol. 2014 Aug;29(8):551-8.

For peer review only

Table S1 Comparison of baseline characteristics between included and excluded patients.

	**LSS**		**LDH**	
	Included	*Excluded*	*Included*	*Excluded*
n	3969	5639	5960	8559
Age, mean (SD)	66 (10.1)	67.8 (10.8)	44.8 (12.6)	44.4 (13.1)
BMI, mean (SD)	27.6 (4.01)	27.7 (4.18)	26.3 (4.12)	26.6 (4.28)
Women, n (%)	1819 (45.8)	2855 (50.6)	2635 (44.2)	3760 (43.9)

Table S2 Distribution of the SF-36 item 11c (I expect my health to get worse) responses preoperatively and year 1 for LSS (n=3969) and LDH (n=5960).

		**1. Definitely True**	**2. Mostly True**	**3. Don't know**	**4. Mostly False**	**5. Definitely False**
LSS, n (%)	*Preop*	78 (1.97)	226 (5.69)	1197 (30.2)	935 (23.6)	1533 (38.6)
	Year 1	157 (3.96)	489 (12.3)	1471 (37.1)	724 (18.2)	1128 (28.4)
LDH, n (%)	*Preop*	93 (1.56)	206 (3.46)	1034 (17.3)	1307 (21.9)	3320 (55.7)
	Year 1	137 (2.3)	427 (7.16)	1483 (24.9)	1270 (21.3)	2643 (44.3)

Table S3 Preoperative and year one postoperative SF-36 data for patients treated surgically for one-level central spinal stenosis between 2007 to 2016.

	**Negative future health expectations preoperatively**			**Positive future health expectations preoperatively**		
	Preop Mean (95% CI)	*Year one Mean (95% CI)*	*SRM (95% CI)*	*Preop Mean (95% CI)*	*Year one Mean (95% CI)*	*SRM (95% CI)*
PF	35 (34;36)	54 (53;55)	0.78 (0.72;0.83)	41 (40;42)	67 (66;68)	1.06 (1.01;1.11)
RP	11 (9.7;12)	38 (36;40)	0.62 (0.57;0.67)	17 (16;18)	57 (56;59)	0.87 (0.82;0.92)
BP	28 (27;28)	50 (49;51)	0.86 (0.82;0.91)	30 (29;31)	60 (59;61)	1.08 (1.04;1.12)
GH	48 (47;48)	53 (51;54)	0.27 (0.22;0.32)	69 (69;70)	69 (68;69)	-0.024 (-0.063;0.015)
VT	35 (34;36)	49 (47;50)	0.59 (0.54;0.64)	44 (44;45)	61 (60;62)	0.68 (0.64;0.72)
SF	53 (52;54)	71 (69;72)	0.61 (0.56;0.67)	63 (62;64)	82 (81;83)	0.66 (0.62;0.71)
RE	35 (33;37)	57 (55;59)	0.42 (0.37;0.47)	52 (50;54)	75 (74;77)	0.46 (0.42;0.5)
MH	61 (60;62)	70 (69;71)	0.45 (0.4;0.5)	71 (70;72)	80 (79;81)	0.44 (0.4;0.48)

Table S4 Preoperative and year one postoperative SF-36 data for patients treated surgically for one-level disk herniation between 2007 to 2016.

	**Negative future health expectations preoperatively**			**Positive future health expectations preoperatively**		
	Preop Mean (95% CI)	*Year one Mean (95% CI)*	*SRM (95% CI)*	*Preop Mean (95% CI)*	*Year one Mean (95% CI)*	*SRM (95% CI)*
PF	37 (36;38)	71 (69;72)	1.2 (1.1;1.3)	41 (41;42)	81 (81;82)	1.54 (1.49;1.58)
RP	8.9 (7.8;10)	53 (51;56)	0.99 (0.93;1.1)	10 (9.7;11)	69 (68;71)	1.37 (1.32;1.41)
BP	20 (19;21)	56 (55;58)	1.3 (1.2;1.3)	23 (22;23)	67 (66;67)	1.55 (1.51;1.59)
GH	49 (48;50)	57 (56;59)	0.39 (0.34;0.45)	74 (74;75)	76 (75;76)	0.076 (0.048;0.11)
VT	28 (27;29)	51 (49;52)	0.88 (0.81;0.94)	35 (35;36)	64 (63;64)	1.1 (1.1;1.1)
SF	42 (41;44)	75 (74;77)	1 (0.97;1.1)	49 (48;50)	87 (86;87)	1.2 (1.1;1.2)
RE	34 (32;36)	68 (66;70)	0.67 (0.61;0.73)	50 (49;52)	83 (82;84)	0.66 (0.62;0.69)
MH	52 (51;53)	69 (68;70)	0.74 (0.68;0.79)	64 (63;65)	81 (80;81)	0.79 (0.75;0.82)

For peer review only

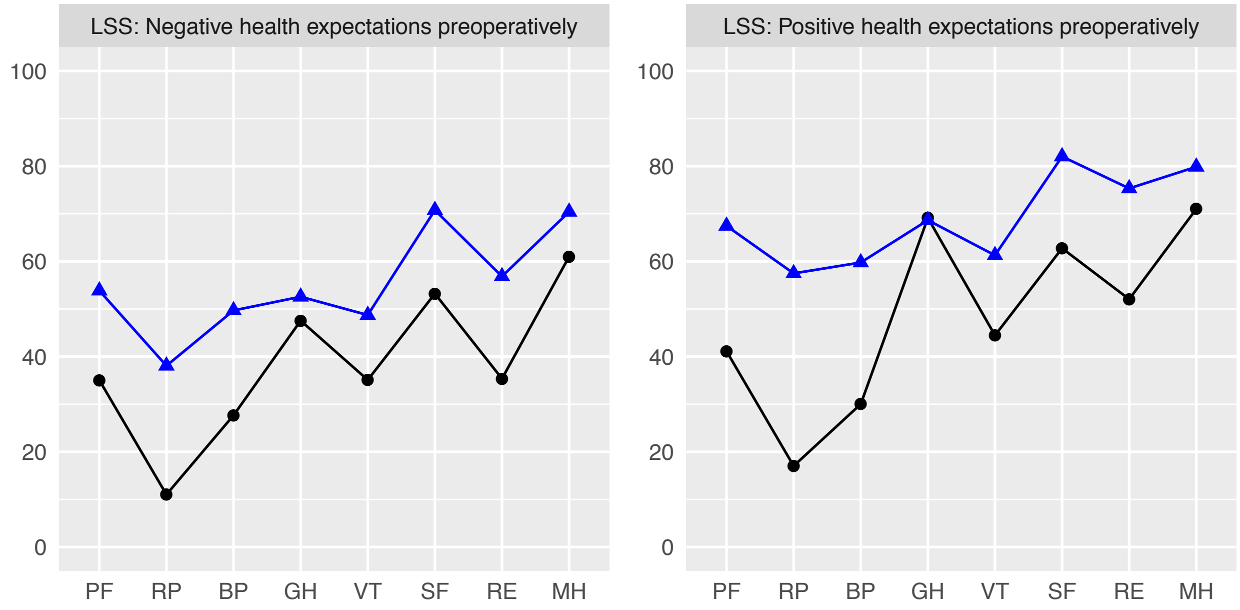

Figure S1 SF-36 profiles preoperatively (black circles) and year one postoperatively (blue triangles) for patients with negative and positive future health expectations preoperatively treated for LSS.
PF = physical functioning, RP = role limitation due to physical problems, BP = bodily pain,
GH = general health, VT = vitality, SF = social functioning, RE = role limitations due to emotional problems, and MH = mental health.

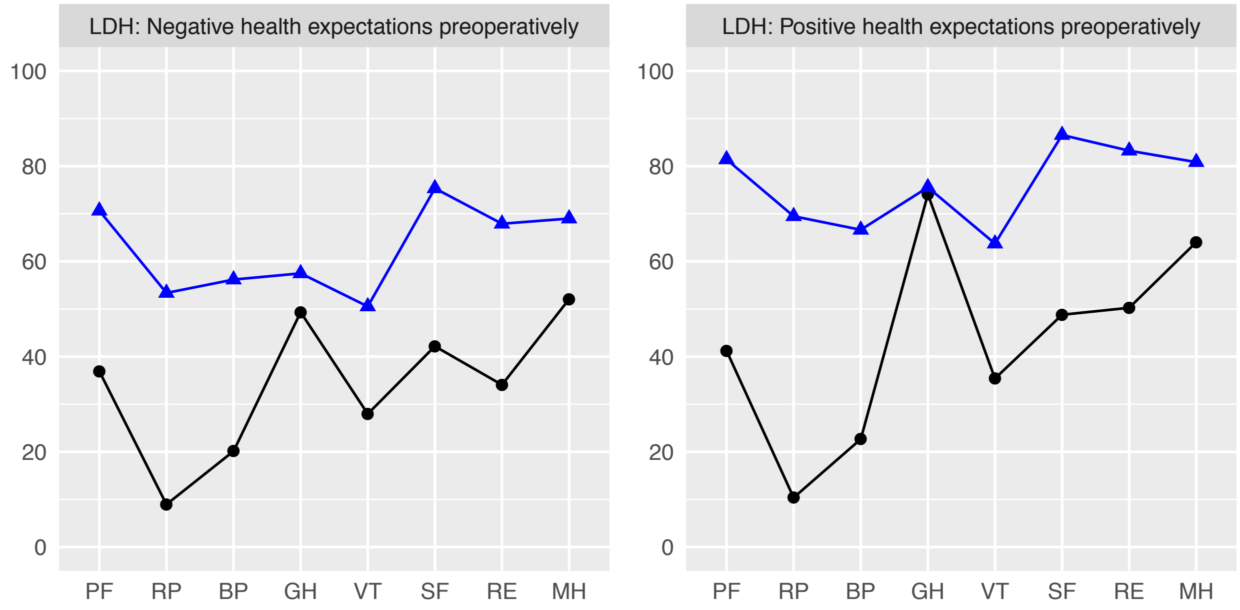

Figure S2 SF-36 profiles preoperatively (black circles) and year one postoperatively (blue triangles) for patients with negative and positive future health expectations preoperatively treated for LDH.
PF = physical functioning, RP = role limitation due to physical problems, BP = bodily pain,
GH = general health, VT = vitality, SF = social functioning, RE = role limitations due to emotional problems, and MH = mental health.

STROBE 2007 (v4) Statement—Checklist of items that should be included in reports of *cohort studies*

Section/Topic	Item #	Recommendation	Reported on page #
**Title and abstract**	1	(*a*) Indicate the study's design with a commonly used term in the title or the abstract	2
		(*b*) Provide in the abstract an informative and balanced summary of what was done and what was found	2
**Introduction**			
Background/rationale	2	Explain the scientific background and rationale for the investigation being reported	4
Objectives	3	State specific objectives, including any prespecified hypotheses	4,5
**Methods**			
Study design	4	Present key elements of study design early in the paper	5
Setting	5	Describe the setting, locations, and relevant dates, including periods of recruitment, exposure, follow-up, and data collection	5
Participants	6	(*a*) Give the eligibility criteria, and the sources and methods of selection of participants. Describe methods of follow-up	5
		(*b*) For matched studies, give matching criteria and number of exposed and unexposed	NA
Variables	7	Clearly define all outcomes, exposures, predictors, potential confounders, and effect modifiers. Give diagnostic criteria, if applicable	5,6
Data sources/ measurement	8*	For each variable of interest, give sources of data and details of methods of assessment (measurement). Describe comparability of assessment methods if there is more than one group	5
Bias	9	Describe any efforts to address potential sources of bias	NA
Study size	10	Explain how the study size was arrived at	5,7
Quantitative variables	11	Explain how quantitative variables were handled in the analyses. If applicable, describe which groupings were chosen and why	7
Statistical methods	12	(*a*) Describe all statistical methods, including those used to control for confounding	7
		(*b*) Describe any methods used to examine subgroups and interactions	NA
		(*c*) Explain how missing data were addressed	7
		(*d*) If applicable, explain how loss to follow-up was addressed	NA
		(*e*) Describe any sensitivity analyses	NA
**Results**			

For peer review only

Participants	13*	(a) Report numbers of individuals at each stage of study—eg numbers potentially eligible, examined for eligibility, confirmed eligible, included in the study, completing follow-up, and analysed	7
		(b) Give reasons for non-participation at each stage	NA
		(c) Consider use of a flow diagram	NA
Descriptive data	14*	(a) Give characteristics of study participants (eg demographic, clinical, social) and information on exposures and potential confounders	7, Table S1
		(b) Indicate number of participants with missing data for each variable of interest	7
		(c) Summarise follow-up time (eg, average and total amount)	7
Outcome data	15*	Report numbers of outcome events or summary measures over time	NA
Main results	16	(*a*) Give unadjusted estimates and, if applicable, confounder-adjusted estimates and their precision (eg, 95% confidence interval). Make clear which confounders were adjusted for and why they were included	7-9, Tables 3-4
		(*b*) Report category boundaries when continuous variables were categorized	NA
		(*c*) If relevant, consider translating estimates of relative risk into absolute risk for a meaningful time period	NA
Other analyses	17	Report other analyses done—eg analyses of subgroups and interactions, and sensitivity analyses	NA
**Discussion**			
Key results	18	Summarise key results with reference to study objectives	9
**Limitations**			
Interpretation	20	Give a cautious overall interpretation of results considering objectives, limitations, multiplicity of analyses, results from similar studies, and other relevant evidence	9-12
Generalisability	21	Discuss the generalisability (external validity) of the study results	9-12
**Other information**			
Funding	22	Give the source of funding and the role of the funders for the present study and, if applicable, for the original study on which the present article is based	13

*Give information separately for cases and controls in case-control studies and, if applicable, for exposed and unexposed groups in cohort and cross-sectional studies.

Note: An Explanation and Elaboration article discusses each checklist item and gives methodological background and published examples of transparent reporting. The STROBE checklist is best used in conjunction with this article (freely available on the Web sites of PLoS Medicine at http://www.plosmedicine.org/, Annals of Internal Medicine at http://www.annals.org/, and Epidemiology at http://www.epidem.com/). Information on the STROBE Initiative is available at www.strobe-statement.org.

For peer review only
